# Medical News

**Published:** 1851-07

**Authors:** 


### MEDICAL NEWS.

Dr. Thos. Stewardson, formerly of this city, has received the appointment of Professor of Natural Sciences in the Georgia Military Institute, situated at Marietta. Dr. Stewardson's early devotion to these sciences, especially Botany, of which his knowledge is extensive, highly qualifies him for such a position. At one time when a resident in this, his native city , his zeal in behalf of Natural History induced him to perform the duties of secretary of the Academy of Natural Sciences, from which his public and private engagements only forced him to return. As a citizen of another state, where health induced him to take up his abode some years since, his learning and acquirements have been appreciated, and we rejoice in the tribute to merit which is evinced by this appointment.

New-York Medical College.—Dr. J. M. Carnochan has been appointed professor of Surgery, in place of Dr. A. L. Cox, resigned; and Dr. Edmund R. Peaslee, of Dartmouth College, Professor of Physiology, Pathology, and Microscopy.

Buffalo Medical College. Some changes have recently taken place in the organization of this Institution. Prof. B. R. Palmer, of Woodstock, Vt., has been appointed to the chair of Anatomy, vacated by the resignation of Prof. Webster, announced in a former number of this Journal.

Prof. C. B. Coventry, having tendered his resignation, has been appointed "in consideration of his services in behalf of the College, and as a token of the high respect in which he is held by the Council and Medical Faculty," Emeritus Professor of Physiology and Medical Jurisprudence. Dr. John C. Dalton, jr., of Boston, Mass., has been appointed to the chair of Physiology and Medical Jurisprudence, vacated by the resignation of Prof. Coventry.

Mesmerism. It may be in the recollection of our readers that Sir Philip Crampton, some time since, in order test the powers alleged to be possessed by persons in what is called the clairvoyant state, produced by the mysterious influence of mesmerism, offered to enclose a bank note for £100, in a sealed envelope, which should become the property of any individual who could, while blindfolded and in the mesmeric sleep, tell its date and number. The challenge has been accepted by Mr. Hill H. Hardy.

Early Struggles of Leuret.—M. Leuret, late physician to the Bicêtre, at Paris, whose premature death was recorded in the April number of the Examiner, was a remarkable example of self-denial and perseverance in working his way to professional rank and fame. His father was a baker, and refused to furnish his son with the means of prosecuting his studies beyond the first year. Poor Leuret was compelled to enter the army as a common soldier. After four years' service, chance so far favored him that his regiment was quartered at St. Denis. His officers were kind and exempted him from mounting guard. The whole of the French army at this period was clothed in white, and the pupils at Salpêtrière have never forgotten the puny little soldier in white uniform, but bespattered from head to foot, who walked every day for years, over the muddy causeway of St Denis, to attend the lectures of Esquirol. The privations which he then endured engendered the disease that cut him off at a later period. He used to sell his bread to buy a candle, wherewith to study during the night in the corner of the barrack, and continued this career with unshaken perseverance, until Esquirol, a man as good as he was great, came to his aid by obtaining his discharge, and appointing him to a place in Salpêtrière.

New Mode of Taking Fees.—The Editor of the *Union Medicale*, in an article on medical organization, mentions the following curious manner of taking fees :—A physician who lived in one of the most populous faubourgs of Paris, and who had an enormous practice, never took any fees at all; but twice a year his wife made a circuit among all her husband's patients with a large bag, which she presented to each, saying, "Put in as much as you like, or as much as you can." The physician in question left a considerable fortune as the result of this confiding practice. Another physician is mentioned, who fatigued two horses daily, and whose fee for every one was only one frank (tenpence) a visit. It is to such conduct as this, when two or three men undersell all their professional brethren, that M. Latour in part attributes the depressed condition of the Profession in France.

M. Cazeaux has been elected a member of the French Academy of Medicine, in the Section of Midwifery.

Death of Dr. Bougon.—Letters from Venice, mention the death of Dr. Bougon, the medical attendant of the family of the Duc de Bordeaux. It will be remembered, that at the time of the assassination of the Duc de Berri by Louval, Dr. Bougon was the first medical man who went to his assistance, and immediately sucked the wound, under the impression that the weapon was poisoned. Dr. Bougon, in 1830, followed the royal family of the elder branch of the Capets into exile, and remained with them till his decease. Such an example of truth and fidelity is indeed honorable to the Profession and to mankind. Would to Heaven the instances were more numerous.

Lord Langdale, who recently died in England, where he was long Master of the Rolls, was originally educated for the medical profession.

MEDICAL PRESCRIPTIONS.—Amongst the many notable schemes which the reform mania of the present day has propounded, is that of compelling physicians to indite their prescriptions in English. Petitions are in fact in the course of signature for this very purpose, if we may believe the newspapers. What the motive may be for this meddling with the usages of the profession we cannot conceive, unless it be the impression that a degree of safety is attainable in dispensing from a prescription in vernacular, which is not so from the Latin. Whatever it be, it will be a matter of little moment, as we do not believe that the legislature will interfere in a proceeding which has always worked well; but it is of importance as an evidence of the tendency on the part of the public to meddle with the medical profession, without the least reference to their welfare or convenience.—*Prov. Med. and Surg. Journ.*

MEDICAL DEPARTMENT OF THE ST. LOUIS UNIVERSITY.—Dr. McPheeters has been transferred to the chair of Materia Medica and Therapeutics in this institution, made vacant by the resignation of Dr. Reyburn; Dr. Willis G. Edwards to the chair of Clinical Medicine and Pathological Anatomy; and Dr. Chas. W. Stevens elected to the chair of General and Descriptive Anatomy.

OBITUARY. Died recently, at Kensington, London, Henry Smith, Esq., surgeon, late of Torrington-square. Mr. Smith combined, with the highest practical attainments, an earnest love for physiological pursuits, and was a zealous co-operator with Dr. Marshall Hall in some of his earliest investigations.

PROFESSOR KÖLLIKER. This distinguished histologist last autumn paid a scientific and recreative visit to Holland and Great Britain, in company with Dr. Czermak. From time to time, he expressed his opinions of what he saw, with regard to the subject which he particularly studies, in four letters, dated respectively from Utrecht, Leyden, Edinburgh and London, and addressed to Dr. Von Siebold, his colleague in the editorship of the *Zeitschrift für wissenschaftlichen Zoologie.*

In Utrecht, he appears to have derived great pleasure from his examination of the labors of Harting, Schroeder van der Kolk, and Donders; and he concludes his first letter by observing, that

"Utrecht is a soil where science flourishes so vigorously, that no attempt should be made to transplant it without careful consideration." The letter from Leyden contains an account of his visit to that place and to Amsterdam.

From Holland, Drs. Kölliker and Czermak passed over to Edinburgh, and visited the Highlands, though, as the author says, not with much profit to science, especially as they had lost the opportunity of exploring the sea off the west coast in company with Forbes and Goodsir. On their return to Edinburgh, they spent ten days with Professor Goodsir, and visited the museums and schools of anatomy in that city. Of Professors Syme, Christison, and Simpson, Dr. Kölliker says:—"In the hospital, we less admired the safety than the ease and elegance with which Syme operates The first is found in Germany; but the latter are more rare . . . Christison does all credit to the great name which he has in Germany; he is highly estimable as a man, and is, perhaps, next to Simpson, the most popular physician in Edinburgh. Simpson is incontestably the first obstetrician in Great Britain; he cannot have many equals, and perhaps no superior." The experiments of Dr. Simpson on parturition in swine, after removal of a portion of the spinal cord, are referred to by Dr. Kölliker, who says that, as far as they go, they show that parturition is independent of the action of the cord. In returning from a visit to the Bass Rock, the travellers were reminded, at the village of Whitechapel, twenty miles from Edinburgh, of the state of the study of anatomy in Britain, about twenty years ago, by the ponderous masses of iron trellis-work which were used to protect the graves in the churchyard from the resurrectionists.

After a visit to Glasgow, Drs. Kölliker and Czermak proceeded to London; and Dr. K. speaks in terms of great satisfaction of the reception which he met with from Mr. Quekett, of the Royal College of Surgeons. His collection of microscopic objects he believes to be in no way inferior to Harting's in Holland, but even to surpass it. W. Bowman, Sharpey, Todd, Wharton Jones, Carpenter, Owen, and Paget, receive each a justly deserved tribute. With regard to the Hunterian Museum, Dr. Kölliker thinks that it ought to be made much more available for instruction than it is at present. "So large an institution," he says, "should also be a large school"

for anatomical instruction. The Regent's Park, Zoological Gardens, the British Museum, and the Museum of Economic Geology, were each visited ; and the hippopotamus is honored with a brief notice.

Dr. Kölliker seems to have expressed his honest opinion of the state of anatomical knowledge in the places which he visited, and of the anatomists with whom he came in connection. British *savants* and practitioners may well feel gratified and encouraged by the tribute which he pays to their industry and knowledge.—*London Journ. of Med., May* 1851.

Illness of the Assistant Physicians at the Ward's Island Hospital.—We learn that five of the Assistants have recently suffered at this Institution with Typhus Fever—Drs. Ravenell, Emmett, Forbes, Blacknall, and Kerly. The first three have recovered, and are absent on sick leave. Dr. Blacknall has sufficiently convalesced to return to his duties, and the recovery of Dr. Kerly, (at present ill,) is confidently anticipated by his physicians.

Emigrant's Hospital, Ward's Island.—It is rumored that Drs. Hosack and Wilkes have resigned their places in the Medical Board of this Institution. We have not heard who are to be their successors.

Another Victim of Typhus Fever.—Dr. Ravenhill, another of the House Physicians at Bellevue, died at the Hospital a few days since, and was buried on Sunday last.

Concours—Election of M. Nelaton. M. Nelaton, one of the surgeons to St. Louis, was elected Professor of Surgery to the Faculty of Medicine on Wednesday last, and thus occupies the chair once filled with so much *éclât* by the illustrious Margolin. The election, it must be confessed, has occasioned universal surprise, for public opinion had selected another and more deserving candidate. The final decision was not arrived at without considerable hesitation,—one might almost say, without compromise of principle. It required no less than four different ballotings to arrive at a result. On the two first, the votes were thus distributed :—

For M. Michon, four votes, viz., Bouillaud, Larrey, and Hervez de Chegoin.

For M. Buisson, three, viz., Velpeau, Begin, and Reveillé Parise.

For M. Nelaton, three, viz., Rostan, Denonvilliers, and Langier.

For M. Robert, two, viz., Malgaigne, Gimella.*

On the third balloting, M. Robert's two voters went over to M. Nelaton ; and, on the final *scrutin* between MM. Nelaton and Michon, the former was elected by a majority of seven to five votes.

The election, as I have said, has produced no small degree of surprise and regret, for the superiority of M. Buisson over all the other candidates was incontestable,—and it is seldom that the result of a Concours disappoints the judgment of the public. But, in the present case, it could hardly have been otherwise. M. Buisson was a stranger to the school of Paris, that is to say, pupil, representative, and professor of a rival institution; and his election would have been a virtual acknowldegement of the decadence of Parisian surgery. The schools of Paris and Montpelier have ever been opposed to each other, both in doctrine and educational practice, and *amour propre*,—a sentiment which influences even the most eminent men,—has, in the present instance, given rise to an act of manifest injustice. This opinion is entertained by the vast majority of those who assisted at the Concours, though many allege, in justification, that the judges could not have acted otherwise.

M. Nelaton, the new Professor, is a young man of very considable promise, and, although the Faculty may not have obtained the services of the best candidate, it will, at all events, be certain of possessing one fully competent to fulfil his duties. This is the main argument in favor of election by Concours. You are always sure of securing a man perfectly able to fill his office, though he may not be the *most* able,—a result which our British system often fails to produce.—*Paris correspondent of Med. Times*, 24*th May*, 1851

Kousso—Cod-Liver—Iodine. The recent importation of kousso into England appears to have a beneficial effect on our speculators here, who have reduced the price of the drug by one half. It is still extensively employed, notwithstanding its high price, and continues to sustain its reputation undiminished. Cod-liver oil, I regret to say, is not only excessively dear here, but of destestable quality. Any London house which would establish a depót in Paris, would confer on

* The number of judges in a Concours is fifteen; but three have been compelled to retire from ill health, viz. MM. Roux, Cloquet, and Gerdy.

us a great benefit, and for itself secure handsome profit. Rancid oil fetches five or six shillings a pint, and at that price it is impossible to bring it into general use. M. Quesnville, the celebrated pharmaceutical chemist, is making strenuous efforts to substitute his amygdalaceous solution of iodine for the "genuine pure," but medical men cannot be induced to abandon one of our most efficacious remedies for a compound the therapeutic effects of which are not yet established.—*Ibid.*

Mistake Corrected.—The following correspondence, between a delegate elected from one of the colleges of New-York, and Dr. Hooker, chairman of the committee on Medical Education, of the American Medical Association, will sufficiently explain itself.

New-York, June 3, 1851.

Dr. Hooker:

*Dear Sir*,—In the New-York Medical Gazette of June 1st, it is stated that in your report before the American Medical Association held in Charleston, you "singled out the instance of a college in New York, whose delegate had a seat in the Association, and which had recently conferred a degree upon one of the most notorious and unprincipled Quacks in the whole country, and that, too, with a full knowledge of his past history and infamous character." A similar statement also appeared in the New-York Register of the same date.

If I am correctly informed, there were but two of the Medical Colleges of New-York represented in the Association.

As a delegate elected from one of these colleges, I would respectfully inquire of you *which* college was thus denounced, and *who* was the individual thus characterized?

I would respectfully submit, that justice demands an explicit answer, inasmuch as the charge bears an official character.

I remain, in haste, respectfully yours, B. Fordyce Barker.

Norwich, June 5, 1851.

Dr. Barker;

*Dear Sir*, Your note of the 3d instant was duly received, and I hasten to reply to it.

The statement to which you refer was *not* made in the report which I presented to the Association in Charleston. The committee indulged in *no such* personality as is *attributed* to them by the

Gazette and Register. When you come to see the report in print, you will find that rumor, as is too often the case, has committed a very *material misrepresentation.*

Yours respectfully, W. HOOKER.

[It will be recollected that we expressed a doubt whether the report of "A Delegate," in our last, was accurately stated; but knowing the source whence we received it, we had no reason to imagine any "misrepresentation." Dr. Hooker and he must settle the matter.]—*N. Y. Med. Gaz.*

SIXTH MEETING OF THE ASSOCIATION OF MEDICAL SUPERINTENDENTS OF AMERICAN INSTITUTIONS FOR THE INSANE.

The following are the propositions referred to in our report of the proceedings of this body in the June No. which were postponed for want of room.

Dr. Kirkbride, from the standing committee on the construction of Hospitals for the Insane, in compliance with the resolution adopted last year, read a report, containing a "series of Resolutions or Propositions, affirming the well ascertained opinions of this body in reference to the fundamental principles which should regulate the erection and internal arrangements of American Hospitals for the Insane."

I. Every Hospital for the Insane should be in the country, not within less than two miles of a large town, and easily accessible at all seasons.

II. No Hospital for the Insane, however limited its capacity, should have less than fifty acres of land, devoted to gardens and pleasure grounds for its patients. At least one hundred acres should be possessed by every State Hospital, or other Institution for 200 patients, to which number these propositions apply, unless otherwise mentioned.

III. Means should be provided to raise ten thousand gallons of water, daily, to reservoirs that will supply the highest parts of the building.

IV. No Hospital for the Insane should be built, without the plan having been first submitted to some Physician or Physicians, who have had charge of a similar establishment, or are practically acquainted with all the details of their arrangements, and received his or their full approbation.

V. The highest number that can with propriety be treated in one building is two hundred and fifty, while two hundred is a preferable maximum.

VI. All such buildings should be constructed of stone or brick, have slate or metallic roofs, and as far as possible be made secure from accidents by fire.

VII. Every Hospital, having provision for two hundred or more patients, should have in it at least eight distinct wards for each sex,—making sixteen classes in the entire establishment.

VIII. Each ward should have in it a parlour, a corridor, single lodgings for patients, an associate dormitory, communicating with a chamber for two attendants; a clothes room, a bath room, a water closet, a dining room, a dumb waiter, and a speaking tube leading to the kitchen or other central part of the building.

IX. No apartments should ever be provided for the confinement of patients, or as their lodging rooms, that are not entirely above ground.

X. No class of rooms should ever be constructed, without some kind of window in each, communicating directly with the external atmosphere.

XI. No chamber for the use of a single patient should ever be less than eight by ten feet, nor should the ceiling of any story occupied by patients be less than twelve in height.

XII. The floors of patients' apartments should always be of wood.

XII. The stairways should always be of iron, stone, or other indestructible material, ample in size and number, and easy of ascent, to afford convenient egress in case of accident from fire.

XIV. A large Hospital should consist of a main central building with wings.

XV. The main central building should contain the offices, receiving rooms for company, and apartments entirely private, for the Superintending Physician and his family, in case that officer resides in the Hospital building.

XVI. The wings should be so arranged, that if rooms are placed on both sides of a corridor, the corridors should be furnished at both ends with moveable glazed sashes for the free admission of both light and air.

XVII. The lighting should be by gas, on account of its convenience, cleanliness, safety, and economy,

XVIII. The apartments for washing clothing, &c., should be detached from the Hospital building.

XIX. The drainage should be under ground, and all the inlets to the sewers should be properly secured to prevent offensive emanations.

XX. All Hospitals should be warmed by passing an abundance of pure fresh air from the external atmosphere, over pipes or plates, containing steam under low pressure, or hot water, the temperature of which at the boiler does not exceed 212 degrees F., and placed in the basement or cellar of the building to be heated.

XXI. A complete system of forced ventilation, in connection with the heating is indispensable to give purity to the air of a hospital for the Insane, and no expense that is required to effect this object thoroughly, can be deemed either misplaced or injudicious.

XXII. The boilers for generating steam for warming the building should be in a detached structure, connected with which may be the engine for pumping water, driving the washing apparatus, and other machinery.

XXIII. All water closets should as far as possible be made of indestructible materials—be simple in their arrangement, and have a strong downward ventilation connected with them.

XXIV. The floors of bath rooms, water closets, and basement stories should as far as possible be made of materials that will not absorb moisture.

XXV. The wards for the most excited class should be constructed with rooms on but one side of a corridor, not less than ten feet wide, the external windows of which should be large, and having pleasant views from them.

XXVI. Wherever practicable, the pleasure grounds of a Hospital for the insane should be surrounded by a substantial wall so placed as not to be unpleasantly visible from the building.

Which propositions having been duly read and maturely considered, were adopted by the Association.

On motion of Dr. Hanbury Smith, it was

*Resolved,* That the Secretary be instructed to cause the propositions now adopted, in reference to the construction and arrangements of Hospitals for the insane, to be published in the Medical Journals of this Continent, as the Sentiments of this Association, on the subject referred to.

